# Amniotic fluid collected from vaginal birth as a source of stem cells for clinical applications and disease modeling

**DOI:** 10.1093/stcltm/szaf017

**Published:** 2025-06-25

**Authors:** Mallory L Lennon, Amy Frieman, Alyssa K Salazar, Igor Kogut, Ganna Bilousova, Jeffrey G Jacot

**Affiliations:** Department of Bioengineering, University of Colorado Anschutz Medical Campus, Aurora, CO 80045, United States; Department of Dermatology, University of Colorado School of Medicine, Anschutz Medical Campus, Aurora, CO 80045, United States; Department of Bioengineering, University of Colorado Anschutz Medical Campus, Aurora, CO 80045, United States; Department of Dermatology, University of Colorado School of Medicine, Anschutz Medical Campus, Aurora, CO 80045, United States; Department of Dermatology, University of Colorado School of Medicine, Anschutz Medical Campus, Aurora, CO 80045, United States; Department of Bioengineering, University of Colorado Anschutz Medical Campus, Aurora, CO 80045, United States; Department of Pediatrics, Children’s Hospital Colorado, Aurora, CO 80045, United States

**Keywords:** amniotic fluid, congenital heart defects, mesenchymal stem cells, stem cells, vaginal birth

## Abstract

Importance: Amniotic fluid is a promising source of autologous cells for disease modeling, drug screening, and regenerative medicine applications. However, current methods of collecting amniotic fluid are invasive, and samples are limited to pregnancies that require amniocentesis or cesarean section. Objective: The purpose of this study was to determine whether amniotic fluid cells could be isolated and cultured from amniotic fluid collected during vaginal deliveries. Intervention: Amniotic fluid samples were obtained during delivery of 4 neonates, 3 of which had been prenatally diagnosed with hypoplastic left heart syndrome (HLHS) in utero. Adherent amniotic fluid cells were assessed for maternal cell contamination, proliferation rate, surface marker expression, and differentiation potential. Amniotic fluid cells were also reprogrammed to induced pluripotent stem cells (iPSCs) and differentiated into functional cardiomyocytes. Results: Amniotic fluid cells collected from vaginal deliveries showed similar surface marker phenotype and differentiation characteristics to amniotic fluid-derived mesenchymal stem cells collected from amniocentesis and cesarean section. Amniotic fluid cells collected during vaginal births of both neonates with HLHS and one neonate with typical heart geometry could be reprogrammed to iPSCs and differentiated to a cardiac lineage with high efficiency. Conclusions and Relevence: These findings suggest that amniotic fluid collected from vaginal births is a readily available source of patient-specific stem cells for banking, in vitro disease modeling, and regenerative medicine applications.

## Graphical Abstract

**Graphical Abstract:**
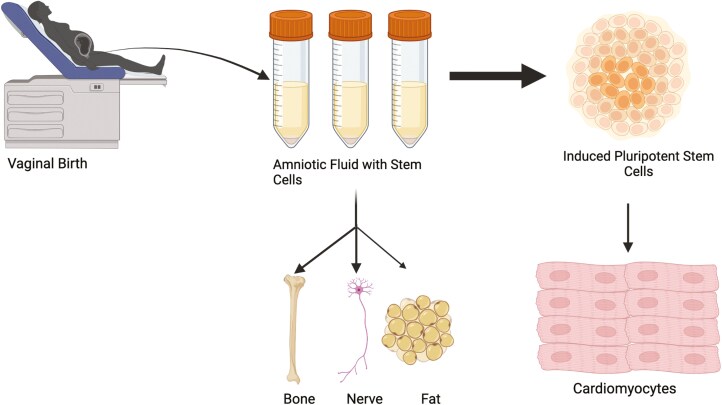

**Significance Statement**

This study found that multipotent stem cells of fetal origin can be isolated from amniotic fluid collected during vaginal deliveries without maternal cell contamination. These cells have marker expression, differentiation potential, and potential for reprogramming to induced pluripotent stem cells similar to amniotic stem cells collected during the second trimester. Cells collected from newborns with structural congenital heart defects and newborns with typical heart structure were differentiated to contractile cardiomyocytes with high efficiency. These results allow for an expanded and readily available source of amniotic stem cells beyond traditional collection through amniocentesis.

## Introduction

Amniotic fluid (AF) is a clear liquid that surrounds the fetus within the amnion, providing mechanical cushioning and a source of nutrients and growth factors to facilitate fetal development.^1^ In addition, AF is known to contain multiple cell types originating from the fetus and fetal membranes.^2^ Amniotic fluid cells (AFCs) have been routinely used as a prenatal diagnostic tool to detect fetal genetic anomalies for decades.^3^ However, following the identification of a subset of proliferative cells with differentiation potential, there has been renewed interest in characterizing AF as a potential source of stem cells for regenerative medicine applications.^4-6^ These studies provided evidence that a portion of mid-term amniotic cells collected during amniocentesis are broadly multipotent. Amniotic fluid mesenchymal stem cells (AF-MSCs) express similar markers to MSCs from other sources, have the potential to differentiate to adipogenic, osteogenic, and chondrogenic lineages, and are easily isolated based on their adherence to plastic.^7^ Later, amniotic fluid stem cells (AFSCs) were isolated based on the expression of the surface antigen c-kit (CD117) and were shown to differentiate into lineages from all 3 embryonic germ layers.^8^

Second-trimester AF-MSCs and AFSCs have now been well characterized, and the fact that they can differentiate into a wide range of lineages, are nontumorigenic, and can be easily isolated with little ethical concerns makes them a promising cell type for therapeutic applications.^5,7,8^ However, collecting AF through amniocentesis has limited the potential of this cell source. Amniocentesis is an invasive procedure and, while routine, is still associated with some risks to the mother and fetus.^9^ In addition, getting samples only from mid-term AF limits access of these cells to certain pregnancies. To address these limitations, recent studies have begun investigating full-term AF as a source of stem cells. These studies demonstrated that both AF-MSCs and AFSCs could be successfully isolated from AF collected during planned cesarean section deliveries.^10-14^ Term AFCs classified as AF-MSCs were highly proliferative and nontumorigenic, expressed mesenchymal surface markers CD105, CD90, CD73, and differentiated to adipocytes, osteocytes, and chondrocytes.^10,11,13,14^ In addition, CD117^+^ AFSCs and unsorted AFCs were both able to develop neural phenotypes.^11,14,15^ These studies showed that collecting AF during full-term cesarean deliveries is feasible, and AFCs isolated from full-term fluid are like those from mid-term AFCs. However, given that less than half of newborns are delivered by cesarean section, collecting samples still poses limitations. The question remains whether AFCs, with similar characteristics to mid-term and cesarean section AF-MSCs and AFSCs, can be isolated during vaginal deliveries.

Cardiac differentiation of CD117^+^ AFSCs has been attempted and published several times with varying methods and varying results, as reviewed by Cananzi and De Coppi.^16^ Several studies have shown that AFSCs can be cardioprotective and even regenerative in rat models through paracrine mechanisms without observed cardiomyocyte (CM) differentiation of AFSCs^17,18^ along with one study observing a very small population of AFSC-derived CMs after injection in a rat heart.^19^ Other studies showed that coculture of CD117^+^ rat AFSCs with neonatal rat CMs could induce cardiac gene expression, a contractile phenotype, and cardiac-like action potentials synchronous with contacting CMs,^19-21^ though cell coupling through fusion or gap junctions could have possibly contributed to that result. For use in regenerative therapies in neonates where large numbers of CMs are required and coculture with heart tissue is unfeasible, a method of chemical-based differentiation of AFSCs to CMs could be highly significant. However, using a process of Wnt signaling-based differentiation^22^ with primary AFSCs induced the expression of cardiac transcription factors Isl-1 and Nkx-2.5, sarcomeric proteins myosin light chain 2v and cardiac troponin I, and gap junction coupling mediated by Connexin-43 expression, but this process never resulted in organized sarcomeres, spontaneous beating, or action potentials.^23^ In another study, OCT3/4^+^ AFSCs were differentiated using the same modulation of wnt signaling and expressed myosin light chain 2v and cardiac troponin T, yet did not contract or have action potentials, and patch clamping showed diminished calcium channel activity.^24^ Overall, the differentiation of AFSCs to CMs without CM coculture has not been demonstrated, leading us to use a strategy of transforming AFSCs to induced pluripotent stem cells (iPSCs) and then differentiating those cells to CMs.^23,24^

Furthermore, the number of CD117^+^ cells in each sample has been highly variable in our experience. Other heterogeneity has been observed in cells isolated from AF in studies by other groups. For example, Perin et al.^25^ found broad heterogeneity in both the gene expression and protein abundance of germ layer and progenitor cell markers, with only 56% of samples positive for C-kit (CD117) expression and only 25% of samples positive for C-kit protein. Additionally, Bottai et al.^11,26^ and Perin et al.^25^ found only 1 of the 7 third-trimester AFSC lines analyzed expressing CD117.

Therefore, reprogramming AFCs into iPSCs would expand the therapeutic potential of AF as a source of stem cells for regenerative medicine. In addition, generating patient-specific iPSC-derived cell lines from full-term AF would be a valuable resource for in vitro disease modeling of monogenic and complex genetic diseases. iPSC-derived cell models are particularly useful for conditions for which few animal models are available, such as congenital heart defects. Generating patient-specific CMs from full-term AF would not only facilitate the study of disease pathogenesis but would also provide a source of autologous CM for tissue-engineered therapies to treat structural heart defects.

Second-trimester AFCs have been reprogrammed to iPSCs using a variety of methods including lentiviral transduction,^27^ Sendai virus,^28^ and modified mRNA.^29^ Full-term AFCs have also recently been reprogrammed using a lentiviral approach.^13^ The accessibility and availability of AFCs provide an advantage over other commonly used source material for reprogramming, such as fibroblasts. In addition, AFCs have been reported to reprogram to iPSCs with higher efficiency than other somatic cell types.^30^ For iPSC lines to be used clinically, it is important to show that they can be reprogrammed using nonintegrating methods. While mid-term AFCs have been reprogrammed using clinically relevant techniques,^29^ it has not been shown that clinically applicable iPSC lines can be generated from full-term AFCs.

CM differentiation from iPSCs was carried out based on a published protocol using small molecules to modulate Wnt signaling.^22^ This protocol has been extensively validated by multiple groups, including our laboratory, to generate functional CMs from pluripotent stem cells.^29,31-36^ Due to the widespread use and reproducibility of this protocol, no alternative differentiation protocols were tested in this study. The evaluation of AFC-derived iPSC ability to differentiate to CMs using different protocols could be the subject of a future study.

Additionally, the use of a low-oxygen environment during iPSC generation was based on a previous study by 2 of the authors of this paper (I.K. and G.B.), which demonstrated that culturing fibroblasts in 5% O_2_ significantly increased the efficiency of iPSC generation.^37^ This strategy was inspired by the work of Yamanaka’s lab, which showed that iPSC reprogramming efficiency was enhanced at 5% O_2_ compared to higher or lower oxygen fractions.^38^ This low-oxygen culture environment reduces cellular senescence and improves reprogramming consistency, making it particularly effective for generating iPSC lines for clinical and research applications.

The purpose of this study was to show that AFCs can be isolated and cultured from AF collected during a vaginal birth. We characterized term AFCs in terms of their expression of multipotency markers and differentiation potential toward MSC lineages and show that they are similar to AF-MSCs collected from amniocentesis and cesarean section deliveries that we have analyzed in previous published studies. We also demonstrate that these cells can be readily reprogrammed into iPSCs using a clinically relevant method and subsequently differentiated into functional CMs. We then demonstrate the potential of term AF as a cell source for in vitro disease models by comparing the properties of iPSC-derived CMs from patients with hypoplastic left heart syndrome (HLHS) and one infant with typical heart geometry.

## Materials and methods

### AF collection

AF samples were collected from Children’s Hospital Colorado with approval from the Colorado Multiple Institutional Review Board (COMIRB #17-2296). Informed consent was obtained from all study participants. AF was collected during delivery after consent had been obtained. HLHS pregnancies were identified after the diagnosis of HLHS in utero. The one sample from an infant was identified as having a structurally normal heart prenatally, with normal heart function confirmed postnatally. AF was collected in a 50 mL syringe from the vaginal canal, if there was spontaneous rupture of membranes, or from fluid that had pooled in a bed pan, if there was artificial rupture of membranes. Maternal DNA was collected after consent using a saliva self-collection kit following the manufacturer’s instructions (OGR-600, Genotek). Newborn DNA was collected using a buccal swab kit for pediatrics following the manufacturer’s instructions (OC-175, Genotek).

### Cell isolation and culture

AF samples were stored at 4 °C and processed within 5 hours of collection. Separation of mononuclear cells from red blood cells was performed using Ficoll-Paque (GE Healthcare). AF samples were diluted 1:1 in RPMI-1640 and filtered through a 100 µm pore size Steriflip filter (Millipore Sigma). Diluted AF was layered on top of Ficoll-Paque in a 50 mL tube and centrifuged at 400*g* for 40 minutes at 20 °C. Cells were resuspended in a modified α-Minimum Essential Medium as previously described,^23^ plated on 0.1% gelatin-coated plates, and cultured at 37 °C and 5% CO_2_. The medium was changed every 2 days. Once cells reached approximately 75% confluency, they were passaged using Accutase (Innovative Cell Technologies).

### Maternal cell contamination

DNA was isolated using the QIAmp DNA MiniKit (Qiagen) from 4 different sources: AFCs, iPSCs reprogrammed from AFCs, fetal buccal swabs, and maternal saliva kits. All DNA samples were provided to the Colorado Molecular Correlates Laboratory to test for maternal cell contamination (MCC). Short tandem repeat testing was performed using the GenePrint 24 System (Promega) according to the manufacturer’s instructions. The results were analyzed using ChimerMarker V3.1.5 software (SoftGenetics) using the single donor chimerism application.

### Flow cytometry

AFCs and human bone marrow-derived mesenchymal stem cells (BM-MSCs; Cyagen) were analyzed for mesenchymal stem markers using flow cytometry. Briefly, approximately 80% confluent cells (P5-P6) were dissociated with Accutase and labeled with CD11b (BD Biosciences, 561001), CD19 (BD Biosciences, 560994), CD34 (BD Biosciences, 560941), CD45 (BD Biosciences, 560976), CD73 (BD Biosciences, 561014), CD90 (BD Biosciences, 5555595), CD105 (BD Biosciences, 562408), CD117 (ThermoFisher, 12-1178-42), CD133 (BioLegend, 372803), and HLA-DR (BD Biosciences, 560944) for 30 minutes in 1% fetal bovine serum (FBS) in phosphate-buffered saline (PBS). Cells were then washed with 1% FBS in PBS and analyzed using a Gallios 561 (Beckman Coulter). Data analysis was performed using Kaluza software (Beckman Coulter). Each file was analyzed for live singlets by eliminating debris with a scatter gate, doublets with a singlets gate, and DAPI (4′,6-diamidino-2-phenylindole, dihydrochloride) was used to exclude any dead cells. The live populations were then analyzed for positivity using a histogram overlay plot comparing the unstained samples to the stained samples.

### Differentiation to MSC lineages

AFCs were cultured to P7 and differentiated to osteocytes and chondrocytes using StemMACS OsteoDiff medium (Miltenyi) and StemMACS ChondroDiff medium (Miltenyi) according to the manufacturer’s protocol. After 21 days, osteocytes were detected using Alizarin Red S (Sigma). Images of Alizarin Red S staining for osteocytes were analyzed using ImageJ (U.S. National Institutes of Health), and using thresholding of the red channel, the percentage of each field of view was calculated. After 30 days, chondrocyte pellets were fixed in 10% formalin, embedded in paraffin, and sectioned for staining with Alcian Blue (Millipore Sigma). AFCs were cultured to P5 and differentiated to adipocytes using StemMACS AdipoDiff medium (Miltenyi) and MesenCult Adipogenic Differentiation medium (StemCell Technologies). After 21-28 days, adipocytes were identified with staining for Oil Red O.

### Reprogramming AFCs to iPSCs

#### Cell lines and culturing

AFCs were cultured in growth medium EGM-2 comprised of EBM-2 Basal Medium (Lonza) supplemented with EGM-2 SingleQuots (Lonza). iPSCs were cultured in Essential 8 basal medium supplemented with Essential 8 supplement and antibiotics (all from ThermoFisher) or mTeSR1 basal medium supplemented with mTeSR1 supplement (both from StemCell Technologies) and antibiotics (ThermoFisher). All cells were grown at 37 °C in a humidified atmosphere of 5% O_2_ and 5% CO_2_.

#### Transfection experiments

AFCs were cultured until reaching 80% confluency. One day prior to transfection, a tissue culture-treated 6-well plate was coated with defined human recombinant Laminin-521 matrix (ThermoFisher) according to the manufacturer’s instructions. AFCs were lifted with Accutase (StemCell Technologies), plated on the Laminin-coated dish at 50,000 cells per well in EGM-2 medium, and incubated overnight in a low O_2_(5%)/5% CO_2_ tissue culture incubator. The following day, the medium was changed to REGM medium, comprised of REBM (Lonza) supplemented with REGM Renal Epithelial Cell Growth Medium SingleQuots (Lonza) and 200 ng/mL of B18R (Gibco), referred to as “reprogramming medium,” using 1 mL per well of a 6-well dish. Cells were incubated for 1 hour in a low O_2_(5%)/5% CO_2_ tissue culture incubator. All of the remaining transfections and subsequent cell culturing were incubated in a low O_2_(5%)/5% CO_2_ tissue culture incubator. For each medium change in the following transfection regimen, the medium was pre-equilibrated in the same O_2_(5%)/5% CO_2_ tissue culture incubator.

To optimize the reprogramming efficiency and consistency of iPSC generation, we used Laminin-521 (LN521) as the matrix coating for the induction of pluripotency in this study. Laminin-521 was chosen based on its superior performance in previous work by our group,^37^ which demonstrated that LN521 provided more consistent results for fibroblast reprogramming compared to Geltrex, with lower batch-to-batch variability, making it more suitable for both clinical and research applications. LN521 offers several advantages for iPSC culture. It provides a chemically defined, xeno-free environment, ensuring consistency and clinical compliance.^39^ By interacting with the α6β1 integrin, LN521 activates survival pathways such as PI3K/Akt, promoting efficient self-renewal and long-term growth without the need for apoptosis inhibitors.^39^ LN521 is also cost-effective for large-scale expansion, facilitating rapid cell amplification (eg, 10-fold in 4 days) with stable pluripotency, making it ideal for scalable applications.^40^ These properties collectively make LN521 a robust and reliable substrate for iPSC induction, providing reproducibility and efficiency suitable for both research and potential clinical applications. However, we acknowledge as a limitation to this study that we did not attempt induction of pluripotency of AFCs from vaginal birth on Matrigel, though we feel that the results would be similar to the induction of other cells.

All modified mRNA (mod-mRNA) transfections were performed using Lipofectamine RNAiMAX (RNAiMAX) (Thermo Fisher Scientific). RNA and RNAiMAX were first diluted in Opti-MEM I Reduced Serum Medium (Opti-MEM) (Thermo Fisher Scientific). For mod-mRNA, the mod-RNA mix was diluted 5×, and 5 μL of RNAiMAX per microgram of mod-mRNAs was diluted 10× using Opti-MEM. After dilution, these components were incubated for 15 minutes at room temperature (RT). After incubation at RT, transfection mixtures of mod-RNA mix and RNAiMAX were applied to the cell cultures, in reprogramming medium. For transfections of miRNA mimics (m-miRs), a 5 μM (5 pmol/μL) m-miR mix was diluted to 0.6 pmol/μL, and 1 μL of RNAiMAX per 6 pmol of m-miRs was diluted 10× using Opti-MEM. The diluted m-miRNA mix and RNAiMAX were mixed together and incubated for 15 minutes at RT. After incubation at RT, the mod-RNA mix was applied to the cell culture followed by the m-miR mix.

**Figure 1.**
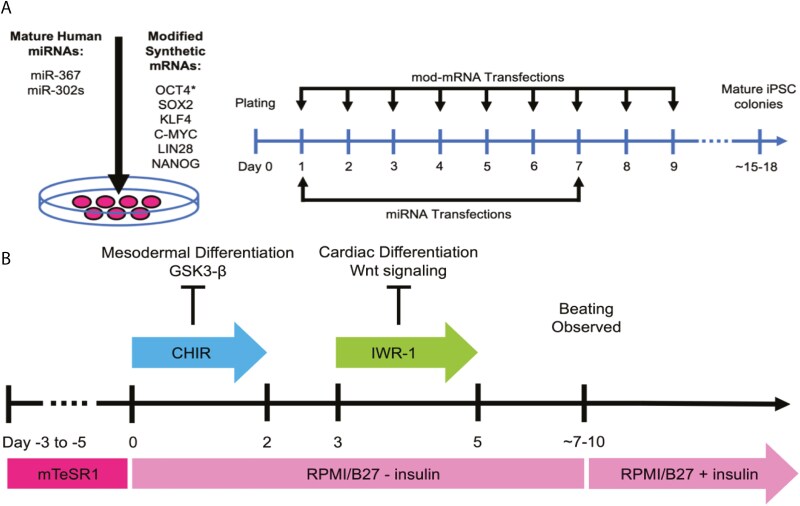
Protocols used in this study. (A) Amniotic fluid cell reprogramming protocol. Amniotic fluid cells were reprogrammed using a 9-day transfection regimen. Cells were transfected every 24 hours with differing amounts of modified mRNAs either alone or in combination with miRNAs. Mature iPSC colonies were observed 15-18 days following the start of reprogramming. (B) Cardiomyocyte differentiation protocol. Reprogrammed AFCs were differentiated to a cardiac lineage using a modified version of a previously published protocol 1. Briefly, iPSC clones were expanded 3-5 days in mTeSR1 before the start of differentiation. On day 0, the medium was changed to RPMI-1640 with B27 supplement minus insulin and 4 μM GSK3 inhibitor (CHIR99021). On day 3, the medium was supplemented with 5 μM Wnt inhibitor (IWR-1). After day 7, cells were maintained in RPMI-1640 with B27 supplement with insulin. Beating cardiomyocytes were typically observed 7-10 days following the start of differentiation. Abbreviations: AFC = amniotic fluid cell; iPSC = induced pluripotent stem cell.

iPSCs were generated using a modified regimen derived from the highly efficient RNA-based reprogramming.^37,41^ The reprogramming mod-mRNA cocktail comprised a modified version of OCT4 fused with the MyoD transactivation domain (called M_3_O) and 5 other reprogramming factors (SOX2, KLF4, cMYC, LIN28A, and NANOG) mixed at a molar ratio of 3:1:1:1:1:1 and included 10% mWasabi mod-mRNA to control for transfection efficiency. The m-miR cocktail included miR-302a, miR-302b, miR-302c, miR-302d, and miR-367 mixed at a 1:1:1:1:1 molar ratio. The detailed procedure for the generation of reprogramming mod-mRNA and m-miR cocktails was previously described.^37,41^ The reprogramming of AFCs consisted of a 9-day transfection regimen (Figure 1A). On day 1, cells were transfected with 100 ng of the reprogramming mod-mRNA cocktail and 10 pmol of the reprogramming m-miR cocktail simultaneously for 4 hours. After 4 hours, medium containing transfection reagents was replaced with fresh, pre-equilibrated reprogramming medium. On day 2, cells were transfected for 4 hours with 300 ng of the mod-mRNA cocktail only, with an equilibrated reprogramming medium change at the 4-hour timepoint. On day 3, cells were again transfected with 300 ng of the mod-mRNA cocktail, allowed to incubate overnight, and reprogramming medium was changed the following morning, day 4. On day 4, cells were transfected with 600 ng of the mod-mRNA cocktail for 4 hours, at which point the reprogramming medium was changed. On day 5, cells were transfected with 600 ng of the mod-mRNA cocktail and incubated overnight, with a reprogramming medium change the following morning, day 5. From day 5 on, “reprogramming medium” consisted of reprogramming REGM supplemented with 200 ng/mL B18R as well as 100 ng/mL human fibroblast growth factor (hFGF) (Gibco). On day 6, cells were again transfected with 600 ng of the mod-mRNA mix and incubated overnight, and the reprogramming medium was changed the following morning. On day 7, cells were transfected with 600 ng of the mod-mRNA cocktail in conjunction with 10 pmol of the m-miR cocktail for 4 hours, at which point the medium was replaced with fresh reprogramming medium. On day 8, cells were transfected with 1 µg of the mod-mRNA cocktail for 4 hours, at which point the reprogramming medium was replaced as on day 7. On day 9, cells were transfected with 1 µg of the mod-mRNA cocktail and incubated overnight. After the last transfection, on day 10, reprogramming medium was changed to pre-equilibrated Essential 8 medium, and the resulting colonies were cultured in Essential 8 medium over the next 5-8 days. Mature colonies were manually picked and replated onto a 6-well format dish precoated with Matrigel (Corning), according to the manufacturer’s instructions into Essential 8 medium. At passage 5, iPSCs were transitioned from Essential 8 medium to mTeSR1 medium and expanded for further characterization.

### Characterization of iPSCs

#### Immunofluorescence

iPSCs were grown on Falcon 4-well Culture Slide precoated with Matrigel (Corning) and a mitomycin C-inactivated human neonatal fibroblast feeder layer. Cells were fixed with 4% paraformaldehyde, permeabilized with 0.02% Triton X (Sigma), and blocked for 2 hours with 10% normal goat serum (Jackson Laboratories) in saponin. Cells were then stained with the following pluripotency-specific antibodies: anti-OCT4 (Santa Cruz Biotechnology, sc 5279: dilution factor 1:100); anti-NANOG (R&D Systems, AF1997: dilution factor 1:50); and anti-TRA-1-81 (Cell Signaling 4745: dilution factor 1:250), and incubated at 4 °C overnight. Secondary antibodies included: Alex Fluor 594 Goat anti-Mouse IgG (H + L) (A-11005), Alexa Fluor 594 Donkey anti-Goat IgG (H + L) (A-11058), and Alexa Fluor 488 Goat anti-Mouse IgG (H + L) (A-11001), and were incubated 2 hours at RT. Stained slides were mounted using a mounting medium with DAPI (Vector Laboratories). Images were captured by the Nikon Eclipse 90i upright microscope using the 10× objective. The brightness and contrast were enhanced using Adobe Photoshop.

#### Karyotyping

Cytogenetic analysis was performed by WiCell using standard GTL banding (G-banding) of metaphase chromosomes. Twenty metaphase chromosome spreads were analyzed for each established line, with chromosome classification following ISCN (2016) guidelines.

#### Teratoma formation

For teratoma analysis, AF-derived iPSCs (3 × 10^6^ cells) were resuspended in a 1:1 mixture of mTeSR1 medium and Matrigel (Corning) and injected subcutaneously into the flank of 6-week-old female SCID or NOD-SCID mice (The Jackson Laboratory). Each of the 4 generated AF-derived iPSC lines were injected into 2 mice. The mice were monitored for 2 months for the formation of a tumor with terminal size limited to 2 cm. If both mice injected with the same iPSC line developed tumors, only one randomly selected tumor was subjected to further analysis. No other randomizations were performed. Investigators were not blinded to the experimental groups. All experiments with mice were performed in accordance with the regulations and approval of the University of Colorado Denver | Anschutz Institutional Animal Care and Use Committee.

#### Quantitative real-time PCR

The expression of embryonic stem cell (ESC) endogenous transcription factors Oct4, TRA-1-81, and Nanog for the AF-derived iPSCs was quantified using quantitative real-time PCR (qRT-PCR) and compared to a human primary neonatal fibroblast line as a control (FN2).^42^ RNA was extracted using the RNeasy Plus Minikit (Qiagen). cDNA was synthesized using the iScriptTM cDNA Synthesis Kit (BioRad).

### Cardiac differentiation

iPSC samples were tested for karyotype stability and mycoplasma infection prior to CM differentiation. This study did not attempt to analyze genetics or mechanism of HLHS, but used these cells to ensure that the iPSC transformation and differentiation to CMs were possible in cells from infants with HLHS. iPSCs were split at a 1:3 ratio using Accutase onto Matrigel (Corning)-coated 12-well plates and cultured in mTeSR (STEMCELL Technologies). After 3 days, iPSCs were differentiated to CMs using a previously described small molecule-based monolayer protocol with minor changes (Figure 1B).^43^ Briefly, on day 0 (start of cardiac induction), medium was changed to RPMI-1640 with B27 supplement (RPMI + B27) minus insulin (Gibco) supplemented with 4 µM CHIR99021 (Selleck Chemicals). On day 2, the media was switched to RPMI + B27 minus insulin. On day 3, medium was changed to RPMI + B27 minus insulin supplemented with 5 µM IWR-1 (Sigma-Aldrich). On day 7, medium was changed back to RPMI + B27 minus insulin. On day 9, media was switched to RPMI + B27 with insulin (Gibco). Medium was changed every 2-3 days from this point forward. Spontaneously beating cells were observed by day 10.

#### Immunocytochemistry

On day 11, iPSC-CMs were plated on Thermanox plastic coverslips (Thermo Fisher) and cultured in RPMI + B27 with insulin for 4 days before fixing. Cells were fixed with 4% paraformaldehyde (PFA), permeabilized with 0.5% Triton X, incubated with mouse anti-cardiac troponin T primary antibody at 1:200 dilution (Thermo Scientific), and detected using Alexa Fluor 546 goat anti-mouse secondary antibody at 1:1000 dilution (Thermo Scientific). Coverslips were mounted with DAPI Fluoromount-G (Southern Biotech). Images were acquired on a Zeiss Axio Observer Z1 inverted phase contrast microscope using Zen Software (Zeiss). A minimum of 300 cells were counted per replicate to determine differentiation efficiency.

#### Quantitative RT-PCR

Cells were harvested for RNA at day 0 and day 30. RNA was extracted using Qiazol Lysis Reagent (Qiagen) and purified using the RNeasy Mini Kit (Qiagen). cDNA synthesis and subsequent qRT-PCR reactions were performed using the High Capacity cDNA Reverse Transcription kit, TaqMan Universal PCR MasterMix, and TaqMan primers (Applied Biosystems). Reactions were performed on the QuantStudio 3 Real-Time PCR System (Applied Biosystems). Data were expressed as relative expression normalized to the housekeeping gene *GAPDH*.

#### Measurement of intracellular calcium transients

iPSC-CMs (day 52 to day 58) were dissociated using Accutase and plated on 25 mm diameter glass coverslips coated with Matrigel. The cells were cultured for 3 days before recording calcium transients. The cells were loaded with 2 µM Fura-2 AM as previously described with a few minor changes.^44^ Pluronic F127 was added at final concentration of 0.01% during loading to improve loading efficiency. Cells were loaded for 10 minutes and washed for 15 minutes with Tyrode’s solution containing (mmol/L): 130 NaCl, 5.4 KCl, 1 MgCl_2_, 0.3 NA_2_HPO_4_, 2 CaCl_2_, 10 HEPES, and 5.5 glucose (adjusted to pH 7.4 with NaOH). Calcium transients were recorded using commercially available software (IonWizard Software, IonOptix). Transients were recorded following the application of field stimulation at 1 Hz, which was approximately 2 times the spontaneous beating frequency. Response to β-adrenergic stimulation was assessed following the addition of 50 µM isoproterenol in Tyrode’s solution.

#### Statistical analysis

Data analyses were presented as mean ± SEM. Statistical analysis was performed using GraphPad Prism 6 Software. The data were analyzed using 1-way analysis of variance (ANOVA) with Bonferroni correction for multiple comparisons. A *P* value <.05 was considered statistically significant.

## Results

### AFCs can be isolated during a natural birth without MCC

AF samples were collected from 4 vaginal births: 3 samples were collected during delivery of neonates that had been diagnosed with HLHS in utero and 1 sample was collected during delivery of a neonate with no structural heart defects (designated as NoDefect) (Supplementary Table 1). All newborns were female, and the average gestational age was 39.1 ± 1.0 weeks. AF samples were obtained following either a spontaneous or artificial rupture of membranes, and AFCs could be isolated and cultured using both collection methods.

**Figure 2.**
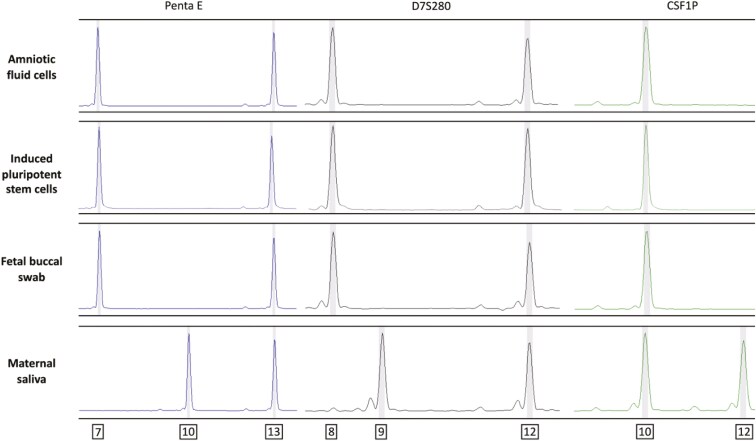
Representative STR analysis (HLHS3 cell line) of 3 informative microsatellite markers used to assess the presence of maternal cell contamination in AF samples collected during vaginal deliveries. For each marker, cells isolated from the AF sample share alleles with reprogrammed AFCs and fetal DNA, but not maternal DNA. Abbreviations: AF = amniotic fluid; AFC = amniotic fluid cell; STR = single tandem repeat.

AF collected using amniocentesis has been shown to contain a level of MCC as high as 62.4% in cultured samples.^45^ To demonstrate that AF from vaginal deliveries is a promising source of autologous cells for regenerative medicine and disease modeling, we wanted to ensure that there was no MCC in cultured samples of AFCs collected during a vaginal birth (VB-AFCs). Short tandem repeat (STR) analysis was used to show that cultured VB-AFCs and iPSCs reprogrammed from VB-AFCs belonged to the newborn. STR analysis results demonstrated that VB-AFCs, AF-derived iPSCs, and fetal buccal swab DNA share a distinct genotype from maternal DNA across all informative alleles (Figure 2). These results confirm that cells from VB-AFC cultures belong to the newborn, and AF collected from vaginal births is a reliable source of cells without MCC. Maternal saliva and newborn buccal swab DNA were collected for HLHS3 and NoDefect deliveries but were not available for HLHS1 or HLHS2 deliveries. However, in HLHS1, HLHS2, VB-AFCs, and iPSC cultures, there were no instances where 3 allele genotypes were observed in one STR loci, indicating that the cells belonged to one donor.

### AF collected at birth is a source of AF-MSCs

**Figure 3.**
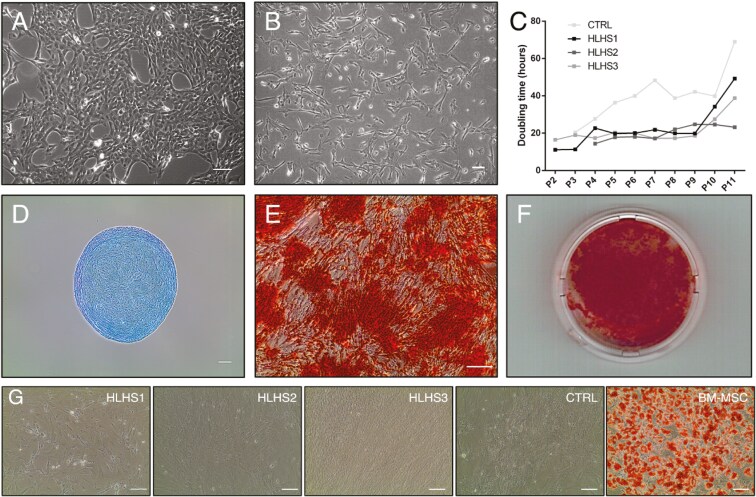
Characteristics of adherent AFCs collected at birth. (A) Representative images showing cell morphology of the primary culture (HLHS2 cell line) and (B) at passage 8 (HLHS2 cell line). Scale bar = 200 μm. (C) Doubling time of VB-AFC lines. Cells were counted every 1-2 days and passaged every 3-4 days. (D) Representative chondrocyte differentiation stained with Alcian Blue (HLHS3 cell line) and (E) osteocyte differentiation stained with Alizarin Red (HLHS3 cell line). Scale bar = 200 μm. (F) 35 mm dish showing a majority of cells staining positive for Alizarin Red (HLHS3 cell line). (G) Adipocyte differentiation of all 4 VB-AFC lines and BM-MSCs stained with Oil Red O. Scale bar = 100 μm. Abbreviations: AFC = amniotic fluid cell; BM-MSC = bone marrow-derived mesenchymal stem cell; VB = vaginal birth.

Adherent cells isolated from VB-AFCs were observed in the primary culture within 9 days of plating (Figure 3A) and displayed a fibroblast-like morphology with passage (Figure 3B). The doubling times were conserved throughout many passages, with all 3 HLHS lines maintaining a doubling time below 25 hours to passage 9 (Figure 3C). The morphology and doubling times of the VB-AFCs were consistent with reports characterizing AF-MSCs collected during second-trimester amniocentesis and cesarean section deliveries.^8,10,46^

To determine whether VB-AFCs exhibit similar differentiation potential to AF-MSCs isolated from second- and third-trimester AF, we assessed the capacity of VB-AFCs to differentiate to 3 mesenchymal lineages: chondrogenic, osteogenic, and adipogenic. Chondrocyte differentiation was tested on one of the 4 lines. Cell pellets were positive for Alcian Blue after culture in chondrogenic conditions for 30 days (Figure 3D). Differentiation to osteocytes was tested in 2 of the 4 VB-AFC lines, as confirmed by positive staining for calcium deposition using Alizarin Red S (Figure 3E). Analysis of images of cells stained with Alizarin Red S in 3 images each across 2 differentiations found that 77 ± 6% of the plates were covered by cells staining red after addition of Alizarin Red. In each differentiation, the majority of the dish area was positive for Alizarin Red S after 24 days in osteogenic medium (Figure 3F). Differentiation of VB-AFCs to adipocytes was not successful in any of the 4 lines. Adipogenic differentiation conditions were applied using 2 different commercially available kits, but no Oil Red O-positive cells were observed after 4 weeks using either method. However, as a positive control, we showed that BM-MSCs stained positive for Oil Red O after just 3 weeks (Figure 3G).

**Figure 4.**
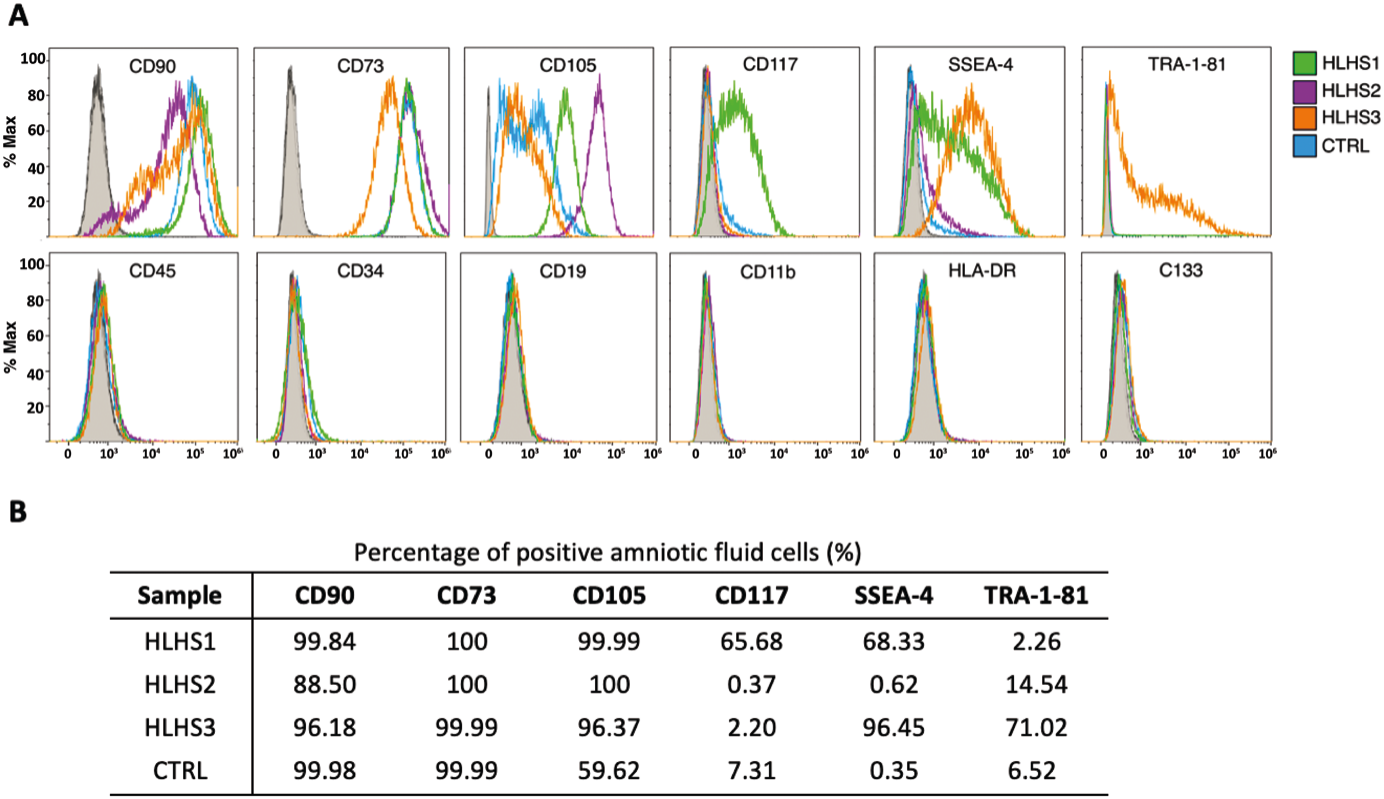
Surface marker expression in VB-AFCs by flow cytometry. (A) Histograms of flow cytometry results for MSC, PSC, and non-MSC markers. Gray histograms represent unstained controls. (B) Percentage of VB-AFCs positive for MSC and PSC surface markers. Abbreviations: AFC = amniotic fluid cell; MSC = mesenchymal stem cell; PSC = pluripotent stem cell; VB = vaginal birth.

Using flow cytometry, we tested whether VB-AFCs expressed surface markers consistent with the criteria for defining an MSC phenotype^47^ and confirmed that all 4 lines were positive for CD90, CD73, and CD105 and lacked expression of CD45, CD34, CD9, CD11b, and HLA-DR (BM-MSCs are shown as a comparison) (Figure 4A). We also analyzed the expression of additional stem cell markers known to be present in some AFC lines isolated during amniocentesis and cesarean section deliveries. The expression levels of the surface markers tested here varied between lines, which is consistent with previous reports.^10,11,25^ Expression of the AFSC marker c-Kit (CD117) ranged from 0.37% in HLHS2 to 65.68% in HLHS1 (Figure 4B). There was also variability in the expression of pluripotency marker SSEA-4, which was expressed in 2 of the 4 lines (HLHS1 and HLHS3) (Figure 4A and B). None of the VB-AFC lines were positive for the multipotent progenitor cell marker CD133^48^ (Figure 4A). Flow cytometry gating strategies for each of the reported values are shown in Supplementary Figure 1.

Using flow cytometry, first we demonstrated the surface marker criteria for defining MSC cultures and confirmed that all 4 lines were positive for CD90, CD73, and CD105 and, as expected, negative for CD45, CD34, CD9, CD11b, and HLA-DR.

### VB-AFCs can be reprogrammed to iPSCs

**Figure 5.**
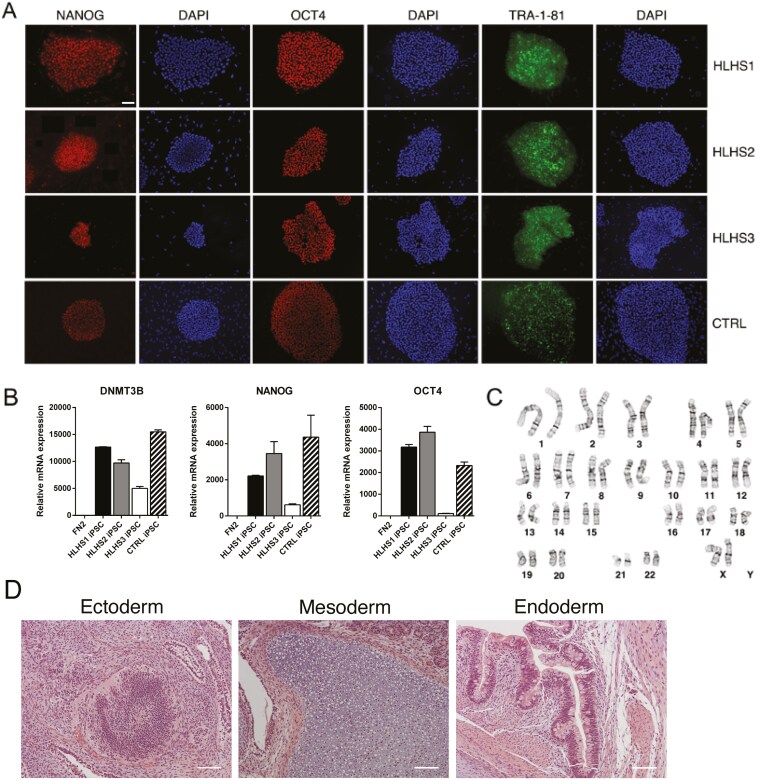
Characterization of iPSC lines derived from VB-AFCs. (A) Immunofluorescent staining for pluripotency markers in all 4 iPSC lines derived from VB-AFCs. Scale bar = 100 µm. (B) qRT-PCR analysis for the expression of pluripotency genes. Relative mRNA expression was normalized to human primary neonatal fibroblast line, FN2 (set to 1), and the housekeeping gene *GAPDH*; *n* = 3 biological replicates. All bars are statistically significantly different compared to FN2 (*P* < .05) (C) Representative example of karyotypic analysis showing a normal 46 XX karyotype at passage 4 (HLHS1 cell line). (D) Representative hematoxylin and eosin stained sections (HLHS1 cell line) of teratomas containing structures specific to ectoderm (neuronal rosettes), mesoderm (cartilage), and endoderm (gut epithelial tissue). Scale bar = 100 µm. Abbreviations: AFC = amniotic fluid cell; iPSC = induced pluripotent stem cell; VB = vaginal birth.

iPSC lines were generated from 4 neonates, 3 with HLHS and 1 with typical heart structure. All lines exhibited molecular and functional characteristics of pluripotent stem cells. Immunofluorescence staining indicated expression of the pluripotency markers NANOG, OCT4, and TRA-1-81 within iPSC colonies (Figure 5A). Quantitative gene expression analysis showed statistically significant upregulation of pluripotency genes, *DNMT3B*, *NANOG*, and *OCT4*, compared to fibroblast controls (*P* > .05). Cytogenic analysis showed normal karyotypes for all iPSC lines (Figure 5C). Subcutaneous injection of iPSCs into immunocompromised mice gave rise to teratomas containing cell types specific to all 3 germ layers (Figure 5D). Together, these results show that fully reprogrammed iPSCs can be derived from VB-AFCs and that iPSC lines can be generated from both healthy and HLHS patients.

### iPSCs derived from VB-AFCs can be differentiated into functional CMs

To show that VB-AFCs could be used for in vitro disease modeling and CM-based therapies for congenital heart disease, we first demonstrated that patient-specific HLHS and NoDefect iPSCs could be differentiated into functional CMs. AF-iPSC lines were differentiated to a cardiac lineage by modulating the Wnt signaling pathway.^49^ Spontaneous beating was observed within 10 days after the start of differentiation in each differentiation performed on each of the AF-derived iPSC lines. A representative video of spontaneous cell contraction of the HLHS1 cell line is included as Supplementary File 1.

**Figure 6.**
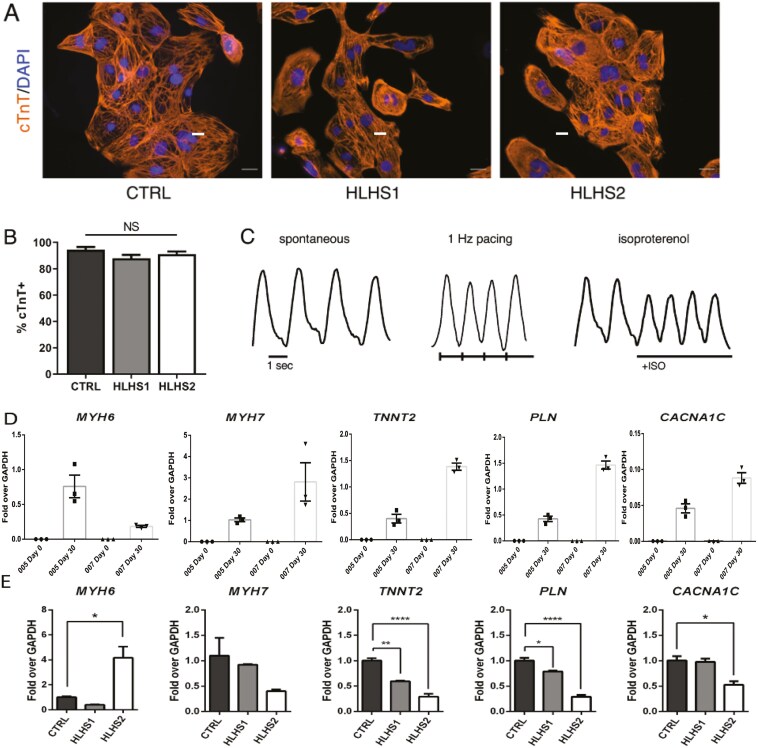
Differentiation of reprogrammed VB-AFCs to CMs. (A) Representative immunofluorescence staining of iPSC-CMs showing cTnT (orange) superimposed with DAPI (blue). Scale bar = 20 µm. (B) Cardiac differentiation efficiency for AF-derived iPSC lines at day 15; *n* = 6 (3 biological repeats × 2 independent differentiations). (C) Calcium transient recording of AF-derived iPSC-CMs from spontaneously beating cells (HLHS2 cell line). (D) qRT-PCR results showing the expression of relevant cardiac genes at day 30 following the start of cardiac induction compared to day 0. (E) qRT-PCR results showing the expression of relevant cardiac genes at day 30 following the start of cardiac induction compared to NoDefect cells. Relative expression was normalized to the housekeeping gene *GAPDH*; *n* = 3 biological replicates. *P < .05, **P < .01, ****P < .0001; NS = not significant in 1-way ANOVA followed by Tukey’s post hoc test. Abbreviations: AF = amniotic fluid; AFC = amniotic fluid cell; CM = cardiomyocytes; iPSC = induced pluripotent stem cell; ISO, isoproterenol; VB = vaginal birth.

At day 21, cells stained positively for the contractile protein cardiac troponin T (cTnT) and sarcomeric structures were visible, indicating successful cardiac differentiation (Figure 6A). iPSCs reprogrammed from VB-AFCs were differentiated to CMs with high efficiency, and the percentage of cTnT-positive cells did not differ between HLHS and NoDefect iPSC-CMs following purification by glucose deprivation (Figure 6B). Calcium transients were recorded in NoDefect iPSC-CMs to assess spontaneous beating rate, response to field stimulation, and chronotropic response to β-adrenergic stimulation (Figure 6C). The cells responded to 1 Hz field stimulation, which was approximately twice the spontaneous beat rate (0.57 ± 0.08 Hz; *N* = 14 cells). In addition, they responded appropriately to β-adrenergic stimulation as evidenced by an increase in beating rate following the addition of β-adrenergic agonist isoproterenol.

Quantitative RT-PCR analysis at day 30 revealed statistically significant upregulation (*P* > 0.05) of cardiac-specific genes implicated in HLHS^28,50^ including *MYH6*, *MYH7*, *TNNT2*, *PLN*, and *CACNA1C* in both the HLHS cell lines as well as the NoDefect iPSC-CMs (Figure 6D). Comparing the 3 lines to each other, we found that the expression of *MYH6* was higher in HLHS2 iPSC-CMs compared to HLHS1 and NoDefect lines, the expression of *TNNT2* and *PLN* was significantly lower in the HLHS1 and HLHS2 lines compared to the NoDefect line, and *CACNA1C* expression was lower in the HLHS2 line compared to the HLHS1 and NoDefect lines. These results demonstrate that AF-iPSCs derived from HLHS and CTRL VB-AFCs can be reliably differentiated toward a cardiac lineage. Quantitative RT-PCR at day 0 (start of cardiac differentiation) showed no significant expression of any of the 5 genes evaluated (*MYH6*, *MYH7*, *TNNT2*, *PLN*, and *CACNA1C*). All values of the expression using the Δ*Ct* method of expression relative to the housekeeping gene *GAPDH* revealed a less than 0.001-fold increase (or less than a thousandth of the expression) over *GAPDH*. The RT-PCR results for one gene, *MYH7*, did not show any amplification in any of the iPSC lines tested.

## Discussion

In this study, we demonstrated that AF collected during vaginal deliveries is a reliable source of AFCs with characteristics similar to those collected during second-trimester and cesarean section deliveries. We successfully isolated AFCs with high expansion potential, confirmed their MSC characteristics, and reprogrammed them into iPSCs. These iPSCs were subsequently differentiated into functional CMs, highlighting their potential for disease modeling and regenerative medicine applications.

Our results showed that AFCs from vaginal deliveries were free of MCC, making them a valuable source for generating patient-specific iPSC lines. The AFCs exhibited surface marker profiles consistent with MSCs, including CD90, CD73, and CD105 expression. Differentiation experiments revealed that these cells could reliably generate chondrocytes and osteocytes but did not successfully differentiate into adipocytes. This finding is consistent with previous reports showing reduced adipogenic differentiation potential in third-trimester AFCs.^26,51^

We also demonstrated that AFC-derived iPSCs could be efficiently differentiated into functional CMs using a Wnt signaling-based protocol.^43,52–54^ These CMs exhibited spontaneous beating, appropriate calcium transients, and pharmacologic responses, confirming their functionality. The study provides a proof of concept for using AFC-derived iPSCs to model congenital heart defects such as HLHS.

### Limitations and future directions

A limitation of this study is the small sample size, which limits our ability to draw robust conclusions regarding differences between HLHS and non-HLHS cell lines. Additionally, we did not demonstrate differentiation of human embryonic stem cells (hESCs) into CMs using the same protocol, although previous studies have validated this protocol with hESCs.^51,52,55^ Future work should include direct comparisons to hESC-derived CMs to further confirm the reproducibility and specificity of the differentiation process.

Future studies should also investigate the potential of AFCs from vaginal deliveries for broader clinical applications, including biobanking, disease modeling, and regenerative therapies. The accessibility and availability of these cells make them a promising source for personalized medicine.

## Conclusion

Collection of AF in connection with vaginal deliveries is feasible and allows for access to a source of cells with similar characteristics to MSCs collected from traditional sources. VB-AFCs can be reliably reprogrammed to a pluripotent stem cell state using a clinically relevant method. The present study also demonstrates that cells from AF collected during full-term vaginal deliveries can be induced to functional, transgene-free CMs from patients with HLHS and from an infant without structural heart defects. The broad applicability and lack of ethical concerns associated with this collection method justify further investigation of this previously underutilized source of cells in biobanking, disease modeling, and regenerative medicine applications.

## Supplementary Material

szaf017_suppl_Supplementary_Material

## Data Availability

The data underlying this article are available at Jacot, Jeffrey (2023), “Amniotic Fluid Collected from Vaginal Birth as a Source of Stem Cells for Clinical Applications and Disease Modeling,” Mendeley Data, V1, doi: 10.17632/rh5npp6zpv.1.
