# Supplementary material for: Amniotic fluid collected from vaginal birth as a source of stem cells for clinical applications and disease modeling
Source: Stem Cells Transl Med. 2025 Jun 25;14(7):szaf017. doi: 10.1093/stcltm/szaf017 (PMC12188528; doi:10.1093/stcltm/szaf017)
Supplement: szaf017_suppl_Supplementary_Material [file szaf017_suppl_supplementary_material.zip › szaf017_Supplemental title page.docx]

Title: Amniotic Fluid Collected from Vaginal Birth as a Source of Stem Cells for Clinical Applications and Disease Modeling

Running Head: Amniotic Fluid Stem Cells from Vaginal Birth

Authors: Mallory L Lennon^1,*^, Amy Frieman^2,*^, Alyssa K. Salazar^1,3^, Igor Kogut^2^, Ganna Bilousova^2,#^, Jeffrey G Jacot^1,4,#^.

Affiliations:

1. Department of Bioengineering, University of Colorado Anschutz Medical Campus, 12705 East Montview Boulevard, Aurora, CO 80045, USA.
2. Department of Dermatology, University of Colorado School of Medicine, Anschutz Medical Campus, 12800 East 19th Avenue, Aurora, CO 80045, USA.
3. Currently at  Department of Biomedical Engineering, The Pennsylvania State University, University Park, PA, 16802, USA.
4. Department of Pediatrics, Children's Hospital Colorado, Aurora, CO 80045, USA.

*,#. These authors contributed equally to this work.

**Author Contributions**

MLL: Collection and assembly of data, data analysis and interpretation, manuscript writing

AF: Collection and assembly of data, data analysis and interpretation, manuscript writing

AS: Collection and assembly of data, data analysis and interpretation

IK: Collection and assembly of data, data analysis and interpretation

GB: Financial Support, data analysis and interpretation

JGJ: Conception and design, financial support, data analysis and interpretation, manuscript writing, final approval of manuscript

**Corresponding author:**

Jeffrey G. Jacot, PhD

12705 E. Montview Blvd, Suite 100

Aurora, CO 80045

Phone: 303-724-8696

[jeffrey.jacot@cuanschutz.edu](mailto:jeffrey.jacot@cuanschutz.edu)

**Research Support:** Research support provided by grants from the National Science Foundation, the National Institutes of Health, Dystrophic Epidermolysis Bullosa Research Association (DEBRA) International, Gates Grubstake Fund, The Gates Institute for Regenerative Medicine, and Children’s Hospital Colorado.

**Key Words:** Amniotic Fluid, Congenital Heart Defects, Mesenchymal Stem Cells, Stem Cells, Vaginal Birth

**Supplementary Information**
