# Supplementary figures and images for: Amniotic fluid collected from vaginal birth as a source of stem cells for clinical applications and disease modeling
Source: Stem Cells Transl Med. 2025 Jun 25;14(7):szaf017. doi: 10.1093/stcltm/szaf017 (PMC12188528; doi:10.1093/stcltm/szaf017)

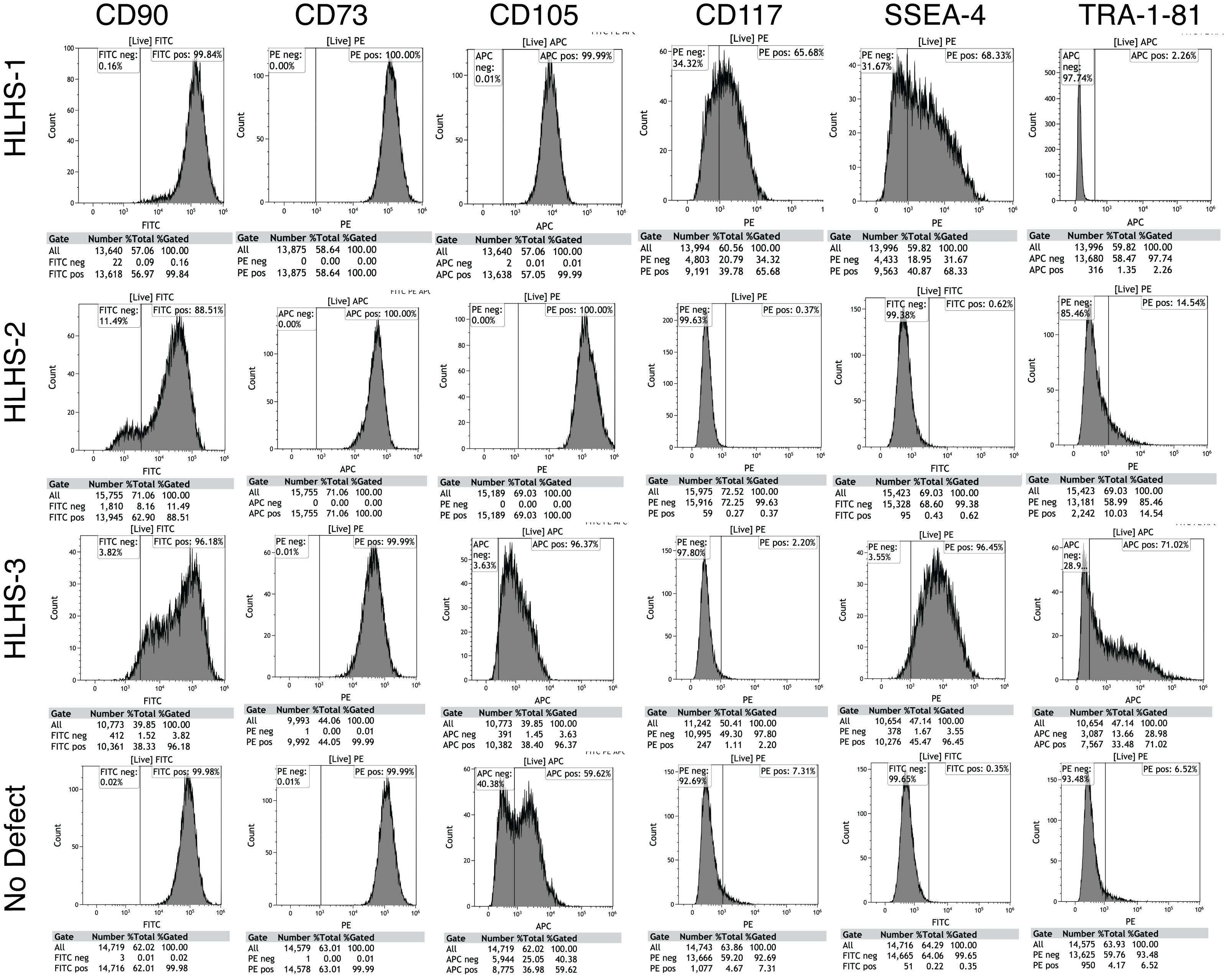

Supplement: szaf017_suppl_Supplementary_Material [file szaf017_suppl_supplementary_material.zip › szaf017_Sup Fig 1.tif]
